# Self-Healing Properties of Bioinspired Amorphous CaCO_3_/Polyphosphate-Supplemented Cement

**DOI:** 10.3390/molecules25102360

**Published:** 2020-05-19

**Authors:** Emad Tolba, Shunfeng Wang, Xiaohong Wang, Meik Neufurth, Maximilian Ackermann, Rafael Muñoz-Espí, Bothaina M. Abd El-Hady, Heinz C. Schröder, Werner E. G. Müller

**Affiliations:** 1 ERC Advanced Investigator Grant Research Group at the Institute for Physiological Chemistry, University Medical Center of the Johannes Gutenberg University, Duesbergweg 6, 55128 Mainz, Germany; emad_nrc@yahoo.com (E.T.); shunwang@uni-mainz.de (S.W.); mneufurt@uni-mainz.de (M.N.); hschroed@uni-mainz.de (H.C.S.); 2 Polymers and Pigments Department, National Research Center, Dokki, Giza 12622, Egypt; bothaina11@yahoo.com; 3 Institute of Functional and Clinical Anatomy, University Medical Center of the Johannes Gutenberg University, Johann Joachim Becher Weg 13, 55099 Mainz, Germany; maximilian.ackermann@uni-mainz.de; 4 Institute of Materials Science (ICMUV), Universitat de València, C/Catedràtic José Beltrán 2, 46980 Paterna, València, Spain; rafael.munoz@uv.es; 5 NanotecMARIN GmbH, Mühlstr. 19, 55218 Ingelheim am Rhein, Germany

**Keywords:** polyphosphate, amorphous calcium carbonate, calcite, Portland cement, microcrack formation, self-healing, 3-point bending, nanoindentation

## Abstract

There is a strong interest in cement additives that are able to prevent or mitigate the adverse effects of cracks in concrete that cause corrosion of the reinforcement. Inorganic polyphosphate (polyP), a natural polymer that is synthesized by bacteria, even those on cement/concrete, can increase the resistance of concrete to progressive damage from micro-cracking. Here we use a novel bioinspired strategy based on polyP-stabilized amorphous calcium carbonate (ACC) to give this material self-healing properties. Portland cement was supplemented with ACC nanoparticles which were stabilized with 10% (*w/w*) Na–polyP. Embedding these particles in the hydrated cement resulted in the formation of calcite crystals after a hardening time of 10 days, which were not seen in controls, indicating that the particles dissolve and then transform into calcite. While there was no significant repair in the controls without ACC, almost complete closure of the cracks was observed after a 10 days healing period in the ACC-supplemented samples. Nanoindentation measurements on the self-healed crack surfaces showed a similar or slightly higher elasticity at a lower hardness compared to non-cracked surfaces. Our results demonstrate that bioinspired approaches, like the use of polyP-stabilized ACC shown here, can significantly improve the repair capacity of Portland cement.

## 1. Introduction

Concrete has proven to be an excellent building material since a few thousand years, like in the Ancient Rome or in the German city of Trier, the Roman “Augusta Treverorum”, with the Basilica of Constantine which was built at the beginning of the 4th century [1]. Even though cement and concrete are often used synonymously, properly termed, cement is an ingredient of concrete. Cement is the binder that adheres to other materials and binds them together [1]. It is an exceptional construction material since it withstands environmental stress and compression but is prone to cracking. The most common cause for early cracks in concrete is plastic shrinkage. Before hardening concrete is in a plastic state and full of water.

Cement, the binder in the concrete, is a powdery substance, composed of calcined lime and clay as the major ingredients [2]. Portland cement is the most common type of cement, which serves as a basic ingredient of concrete, mortar, stucco and grout [3]. The reaction of the silicate phases of Portland cement with water (hydration reaction) results in the formation of calcium silicate hydrate (C–S–H) which constitute the fundamental and the binding phase of concrete. During the setting process (aging/hardening of the cement), most of the sulfate is normally consumed to form ettringite. Cracks in concrete start to develop with microcracks that allow the ingress of water, carbon dioxide and chlorine ions. This process may involve degradation and corrosion of reinforcement resulting in regular and costly repair after a likewise costly maintenance work.

In conventionally reinforced cement/concrete cracking is basically unavoidable since thermal effects, early age shrinkage, mechanical loading, freeze-thaw effects or a combination of these factors influence the stability of the material [4]. Therefore, strong efforts have been undertaken to prevent cracking or to introduce new additives to make the material more resistant. Two repair strategies exist [5]: At first, to exploit the natural autogenous/self-healing properties of the cement (autonomic; without any intervention) and second, to take advantage of the artificial healing capacity of the cement (nonautonomous; needs human intervention/external triggering), based upon the exploitation of embedded adhesive repair agents in the material [6]. It has been proposed that the unreacted cement particles undergo hydration and, by this, forms hydration products which close the gaps. These rehydration products are found in small cracks (1–10 μm) due to freeze–thaw cycles and have been characterized as C–S–H as the main component of the reacted cement paste [7]. Supportive was the finding that cement specimens after reloading and curing in water and air gain the initial stiffness, coinciding with new hydrates being formed [8]. Other studies have provided some evidence that the self-healing process involves the precipitation of calcium carbonate (CaCO_3_) on the crack surface [9,10,11]. The white crystalline material, appearing as coated sheet, is formed either during the carbonation reaction, running with calcium hydrogen carbonate [Ca(HCO_3_)_2_] coming from the water and reacting with calcium hydroxide [Ca(OH)_2_] from concrete or from the calcium hydroxide reacting directly with CO_2_ from the air [10].

We introduced polyphosphate (polyP), a natural inorganic polymer, into the field by showing that this polymer—if added to the cement—provides this material with self-healing properties [12]. PolyP is also synthesized in bacteria, residing in or on the cement—and also in the cracks—has been proposed to initiate and facilitate the self-healing process in cement [12]. The latter process is mediated by a phase transformation of polyP into a functionally (bio)active coacervate state on the surfaces of bacteria [13,14].

In the present study we investigate the self-healing potential of amorphous calcium carbonate (ACC) in cement. The amorphous state of the ACC was stabilized with 10% (*w/w*) of Na–polyP [15] and added as such into the cement samples [12]. The hypothesis was that in the cement ACC undergoes crystallization to crystalline calcite and in parallel polyP is degraded by existing bacteria to calcium phosphate which also crystallizes. The experiments summarized here indicate that indeed addition of stabilized ACC into the cements substantially accelerates the self-healing process of the microcracks. The data suggest that this approach may be suitable for application of the material for conservation of damaged monuments with beginnings of larger damages [16].

## 2. Results

### 2.1. Morphology of Amorphous Calcium Carbonate Nanoparticles

**Figure 1:**
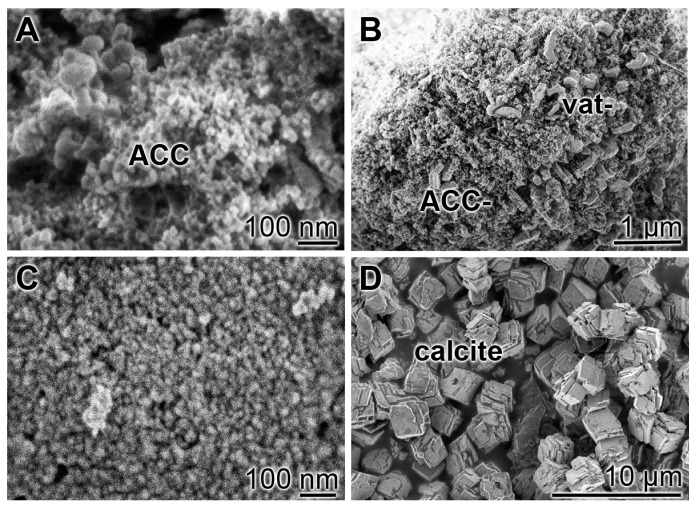
Morphology of amorphous CaCO_3_ nanoparticles (ACC-NP) versus calcite crystals; SEM. The ACC-NP were used for microscopic inspection either (**A**) immediately after precipitation or (**B**) after an aging period of 3 h or after 3 days. (**C**,**D**) In parallel, CaCO_3_ was directly prepared in the absence of polyP; then calcite is formed. Amorphous CaCO_3_ (ACC), vaterite (vat) and calcite particles are marked.

The amorphous calcium carbonate nanoparticles (ACC-NP) were prepared from CaCl_2_•2H_2_O and Na_2_CO_3_. If samples were taken after a short precipitation process of 30 min the preparation had the described spheric morphology of a size of ≈20 nm (Figure 1A), as described [17]. After an aging period of 3 h or 3 days the first vaterite aggregates appeared (Figure 1B,D).

In a separate series of experiments the Ca-carbonate was directly synthesized in the absence of polyP; calcite is formed (Figure 1D).

### 2.2. XRD Analyses of ACC-NP

**Figure 2:**
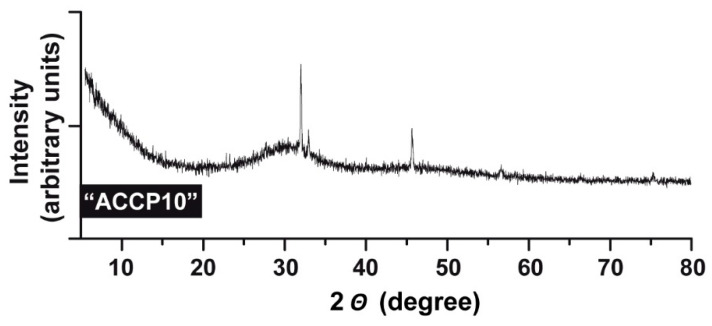
X-ray diffraction analysis. The XRD pattern was obtained from ACC particles stabilized with 10% (*w/w*) Na–polyP, “ACCP10”. The NaCl signals highlight as sharp spikes.

Samples of “ACCP10” were analyzed by XRD and found to be largely amorphous (Figure 2). Only a few signals clearly assigned to NaCl are observed.

### 2.3. Morphology of Cement Supplemented with “ACCP10”; “hCEM•ACCP10”

The commercially obtained, unhydrated cement, “CEM” was supplemented with water and then stirred. The resulting hydrated cement (“hCEM”) slurry was filled into plastic molds (5 cm × 2.5 cm × 2.5 cm) and hardened either for 3 days or 10 days at room temperature in a humidity chamber at 20 °C. Demolding was after 24 h.

**Figure 3:**
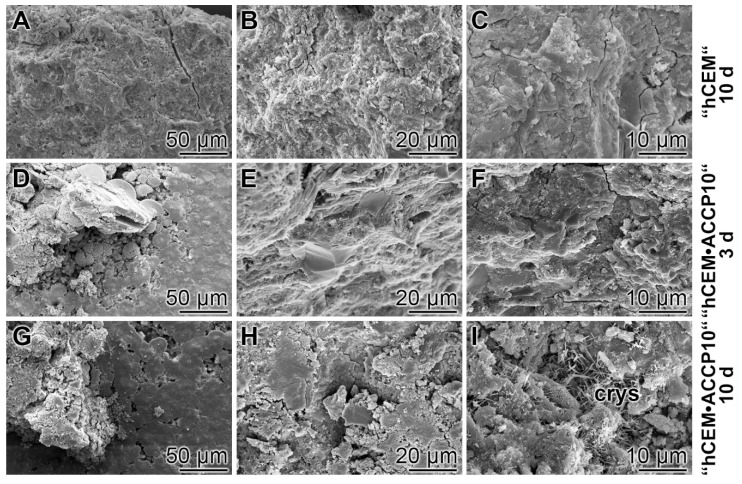
Surface structure of commercially obtained, unhydrated cement supplemented with water (“hCEM”) compared to cement, supplemented with “ACCP10”; ESEM. The (**A**–**C**) non-supplemented cement after processing with water and subsequent hardening for 10 days as well as the cement (**D**–**F**) enriched with “ACCP10”. This “hCEM•ACCP10” formulation did not show prevalent crystal structures (after 3 days). (**G**–**I**) However, the ACC-containing cement samples, hardened for 10 days, frequently showed needle-like reticular C–S–H structures or crystalline needle–fibers (crys).

In the hydrated cement “hCEM” (hardening period, 10 days) and also in the “hCEM•ACCP10”, after a hardening period of 3 days, no crystal structures can be visualized by environmental scanning electron microscopy (ESEM) (Figure 3D–F). However, after 10 days hardening, frequently crystal structures were observed (Figure 3G–I). They appeared in the vicinity of the calcium silicate hydrate (C–S–H) patches [18,19]. The amorphous calcium carbonate, which is present in the cement only in low concentrations (1%), cannot be microscopically resolved; only the calcite crystals are seen.

### 2.4. FTIR Analyses

**Figure 4:**
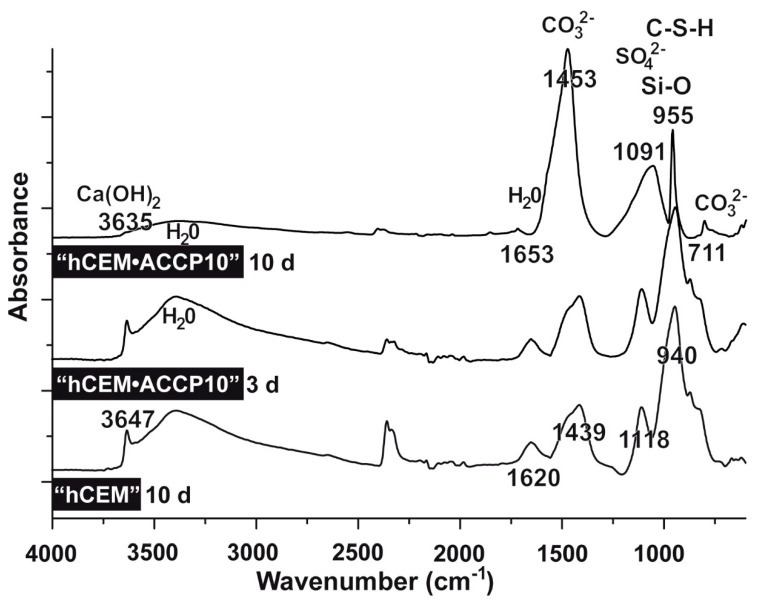
FTIR spectra of hydrated cement, “hCEM”, as well as of the hydrated cement supplemented with “ACCP10”, “hCEM•ACCP10”. The cement samples were incubated for 3 days or 10 days, as indicated. The assignments and the characterization of the peaks of cement, also after processing and aging are discussed in the text. The marked peaks correspond to Ca(OH)_2_, H_2_O, CO_3_^2−^, SO_4_^2−^, Si-O as well as the C–S–H regions and also to CO_3_^2−^. The spectra were recorded between the wavenumbers 4000 and 300 cm^−1^. The absorbance is given in arbitrary units.

The addition of “ACCP10” to the “hCEM” does not change the FTIR spectra markedly (Figure 4). The major absorption bands were found are as follows. The small band at the wavenumber of 3647 cm^−1^ can be assigned to stretching O-H of Ca(OH)_2_ which is one major product during the cement hydration process. The broad band around the region of 3400 cm^−1^ is caused by the stretching vibration of water molecules as well as the band at ≈1650 cm^−1^ which reflects the bending vibration of the adsorbed molecular water. The band around 1450 cm^−1^ corresponds to the stretching vibration of carbonate group and the weak shoulder around 870 cm^−1^ is due to the out-of-plane bending of carbonate group. The band at 1100 cm^−1^ is related to the stretching of SO_4_^2−^ perhaps originating from ettringite and monosulfate. The silicate vibration regions of C–S–H, coming from the silicate polymerization in the presence of water and higher alkaline conditions, is observed at the sharp band at 950 cm^−1^ due to Si-O stretching vibrations of the silicate structure. Only weak shifts are seen for the carbonate peak at around 1450 cm^−1^ between “hCEM” and the “ACCP10”-containing specimens hardened for 3 days or 10 days; this peak increased with progressing incubation time. The intensity of the stretching vibration of SO_4_^2−^ around 1100 cm^−1^, shifts to a lower wavenumber after the addition of “hCEM•ACCP10”. The stretching of Si-O, originating from the silicate structures within the C–S–H complex around the band at 950 cm^−1^, shifts between 955 cm^−1^ and 940 cm^−1^. In addition, the intensity of stretching vibration of the carbonate group changed and a new band appeared at 711 cm^−1^. This change is characteristic for the calcite phase of calcium carbonate and is seen in the spectrum of “hCEM•ACCP10” after 10 days of hardening. Especially the latter change reflects a progressive crystallization of the silicate chains “hCEM•ACCP10” cement in contrast to “hCEM”.

### 2.5. XRD Analyses of Cement Samples

**Figure 5:**
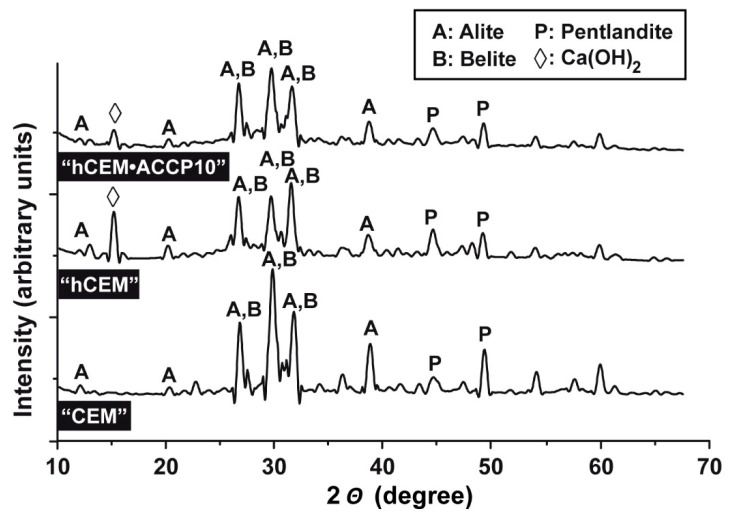
XRD patterns of “CEM”, “hCEM” and “hCEM•ACCP10”. The characteristic signals of alite, belite, pentlandite and Ca(OH)_2_ are marked. The analysis of the hydrated cement samples, “hCEM” and “hCEM•ACCP10”, was performed after an aging period of 10 days.

The calcium silicate phases, tricalcium silicate (alite, C_2_S) and dicalcium silicate (belite, C_3_S), are among the main components of Portland cements and react with water molecules (hydration reaction) to form calcium silicate hydrates [C–S–H]. Upon hydration reaction, calcium hydroxide is also liberated during the process. The XRD spectrum of cement powder shows strong signals in the XRD pattern, especially around the 2*θ* (degrees) of 30 which is attributed to alite and belite crystals (Figure 5) [20]. The same pattern is seen both in “hCEM” and also in “hCEM•ACCP10”. The recorded diffracted signals derived from the cement samples at 2*θ* of 29.5 match with those from the references of C_3_S and C_2_S, at 2*θ* of 30.1 with C_3_S, at 2*θ* of 31 with C_2_S and at 2*θ* of 32.5 and 34.4 with C_3_S and C_2_S [21]. Notable is also the appearance of the Ca(OH)_2_ signal in the cement after addition of water, followed by a change in the pH [22].

### 2.6. Mechanical Properties–Compression Testing

**Figure 6:**
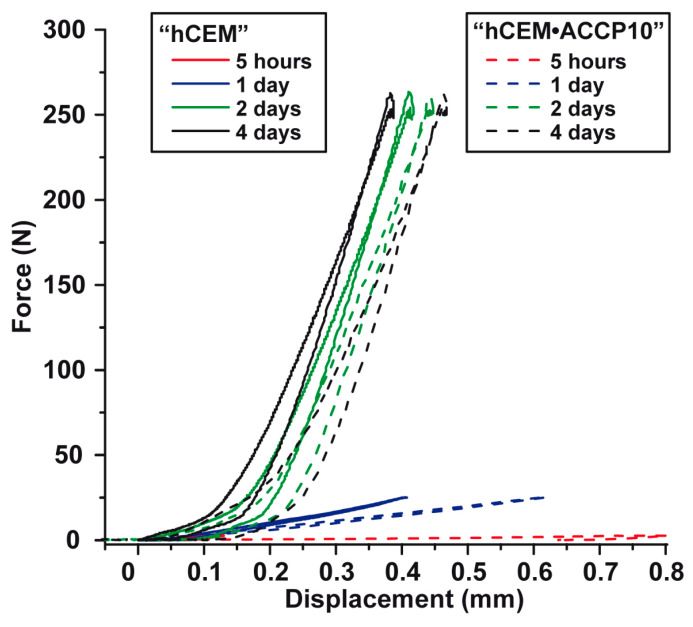
Mechanical properties (load-force) of the cement samples “hCEM” and “hCEM•ACCP10”. After the indicated incubation periods, 5 h (the graphs are close to the abscissa), 1 day, 2 days and 4 days, the displacement of the samples was compared between “hCEM” (solid lines) and “hCEM•ACCP10” (broken lines).

Force/displacement curves were obtained from the compression testing studies both with “hCEM” and with “hCEM•ACCP10” (Figure 6). Altogether addition of “ACCP10” to the cement shows only little effect on the overall compressive strength of the material. However, the impact on the elasticity is marked and it decreases during the incubation time. After 5 h the cement control shows a displacement of 0.13 mm upon loading with 2.5 N, while for the “ACCP10”-enriched cement the maximum displacement after 5 h amounts to 0.8 mm which is 515% higher if compared to the “hCEM” control. After 1 d of incubation the displacement of the control amounts to 0.41 mm upon loading with 25 N, while the displacement of the ACC treated concrete is with 0.62 mm still 51% higher if compared to the untreated control. Two days after mixing the stability of the concrete materials, both for “hCEM” and “hCEM•ACCP10” markedly increased. In parallel, the differences in the elasticity are less pronounced. The untreated control shows a displacement of 0.42 mm upon loading with 250 N and for “hCEM•ACCP10” a value of 0.45 mm; and is with this only about 7% higher if compared to the “hCEM” control. The final measurement after 4 d of incubation shows comparable values. The deformation of the untreated concrete control amounts to 0.39 mm, while “hCEM•ACCP10” cement shows a value which is 21% higher.

### 2.7. Mechanical Testing: 3-Point Bending Studies

**Figure 7:**
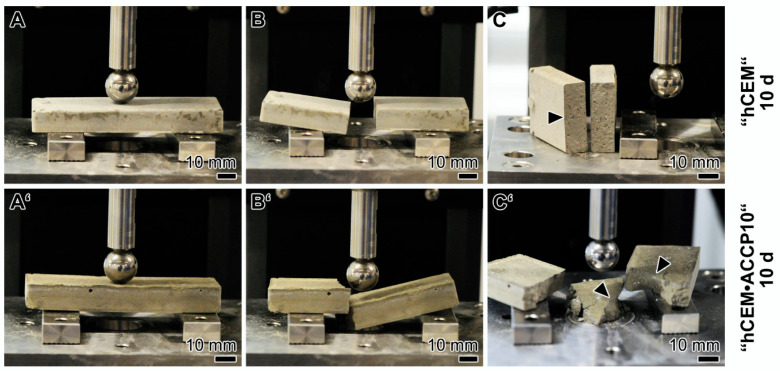
Three-point bending system applied to assess the resistance of the control cement “hCEM” (**A**–**C**) in comparison to the “hCEM•ACCP10” material (**A’**–**C’**). During the bursting phase, a sharp breaking plane is seen in the controls (**C**; arrowhead), while in the “ACCP10”-supplemented material (**C’**; arrowheads) the block bursts into several pieces with different sizes.

**Figure 8:**
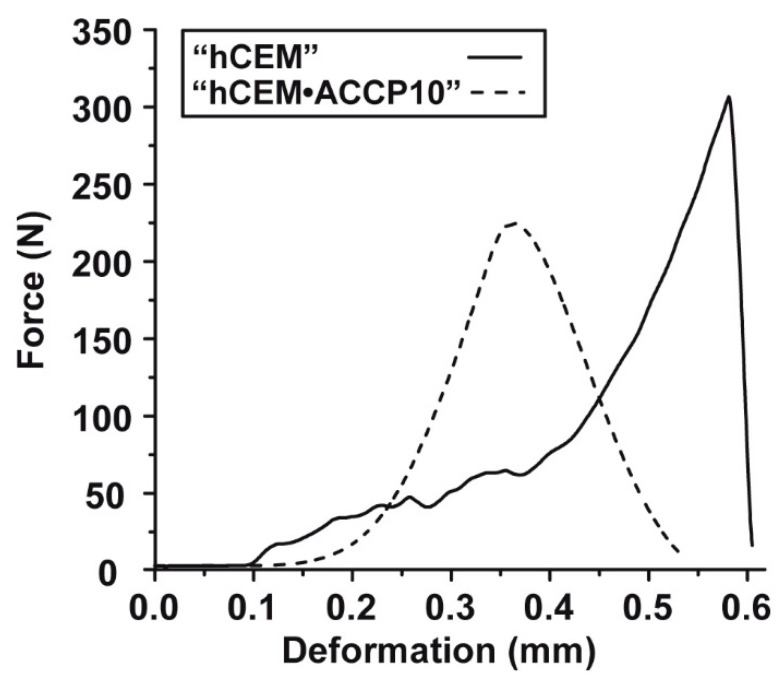
Force–displacement reaction of “hCEM” (solid line) in comparison to the one of the “hCEM•ACCP10” material (broken line).

The breaking load of the Na–polyP/amorphous calcium carbonate (“ACCP10”) doped cement samples was determined and compared with the properties of the “hCEM” control by using the 3-point bend system. The samples were kept for 10 days at room temperature (20 °C). After putting onto the platform of the machine, the samples were exposed to increasing loads until a failure of the materials was detectable. For the cement control, the result of the respective images is depicted in Figure 7A–C. At the beginning of the measurement (about 90 sec) the sample looks intact (Figure 7A). After the first rupture the specimen breaks into to two distinct pieces directly below the applied load (Figure 7B). The broken specimen shows straight breaking edges without noticeable fraying (Figure 7C; arrowhead). This fracture behavior is also reflected by the measured force deformation graph (Figure 8; solid line). After a slowly ascending phase with several slight microcracks which are most probably already created during the casting process, the graph shows a steep increase. Then, after reaching the maximal force at 304 N the material breaks and the force decreases immediately to 0 N. Accordingly, a sharp peak becomes apparent at the breaking point.

For the “hCEM”-enriched cement, “hCEM•ACCP10” (aged for 10 days), the fracture behavior is strikingly different. At the beginning of the measurement, again an intact block is visible (Figure 7A’). After increasing loading the material fails, but the plane of breaking fracture zone is not located below the applied load punch; the fracture surface is displaced outwards (Figure 7B’). Furthermore, this cement material is breaking into several pieces of varying shape and size. The breaking edges are irregularly shaped (Figure 7C’; arrowheads), again without noticeable fraying. After recoding the respective force/deformation graph (Figure 8; dashed line) again an initial short nonlinear phase is seen followed by a steep almost linear increase in the force values until the maximal force of 223 N is reached. In this “ACCP10”-enriched sample a broader breaking point is recorded. After breaking, the graph shows a much smoother decrement. Interestingly, in this descending phase a starting deformation is visible. This latter observation reflects the breakdown of the material into several smaller parts, which is in distinct contrast to the single breaking edge of the cement control sample. In addition, the maximal force is lower for the “hCEM•ACCP10” cement with 223 N, compared to the cement control with 304 N.

### 2.8. Self-healing Potential

The cement samples, either “ACCP10”-free, “hCEM” or cement containing “ACCP10” nanoparticles (“hCEM•ACCP10”) were selected. The samples were aged for 10 days. Microcracks were inserted into 10 days aged cement blocks by shock freeze–warming cycles. The samples, comprising microcracks between ≈15 and ≈66 µm, were submersed in water up to 3 mm below the top edge of the blocks and continued to be left for additional 10 days.

**Figure 9:**
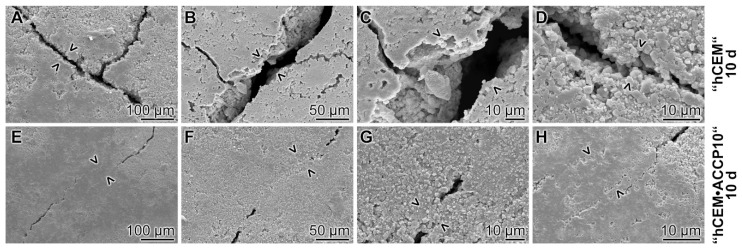
Self-healing potential of “hCEM•ACCP10” cement. Microcracks (> <) were introduced into “hCEM” (**A**–**D**) and into “hCEM” supplemented with “ACCP10” (**E**–**H**) and the samples were subsequently submersed into water for 10 days. (**A**–**D**) While after this period of 10 days in the controls, “hCEM” samples, no closure of the cracks was visible, the gaps in the (**E**–**H**) samples of “hCEM”, supplemented with “ACCP10”, were almost completely repaired; the initial appearance of the cracks at day 0 is marked (> <).

Light microscopic inspection revealed that the cracks induced in the samples showed an irregular progression pattern, following the micrograins within the cement. After treatment in water for additional 10 days the “hCEM” samples were inspected again. Although it is likely that a significant amount of unhydrated cement is still present in the “ACCP10”-free “hCEM” sample after this period of time, no significant change in the morphology of the cracks was seen, if compared to the one at the beginning of the 10 days period (Figure 9A–D). In contrast, almost all cracks were sealed in the “hCEM•ACCP10” samples with a more granular material (Figure 9E–H).

### 2.9. Surface Texture

**Figure 10:**
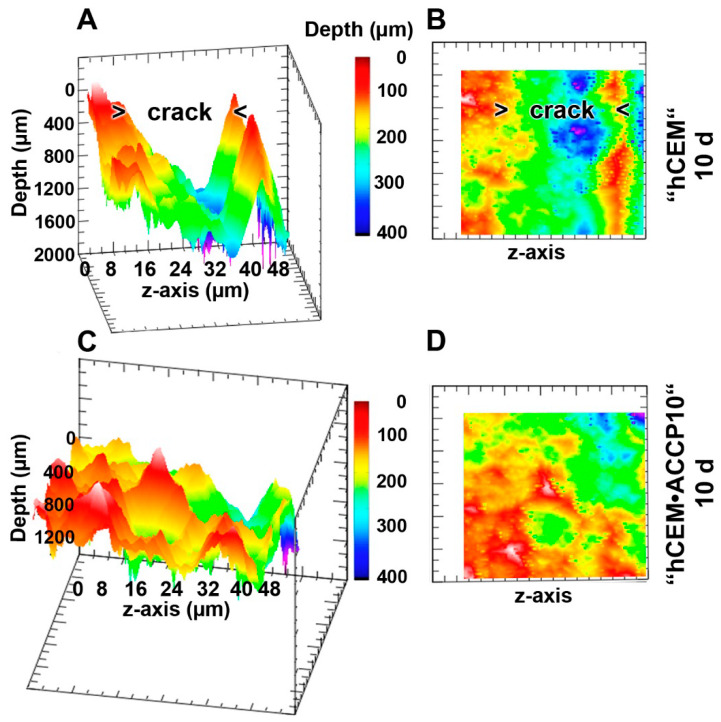
Surface texture image analysis of samples around the crack area taken from (**A**,**B** [corresponding image to **A**]) “hCEM” control cement specimens, aged for 10 days and post-incubated in water for additional 10 days and from (**C**,**D**) polyP-doped samples, “hCEM•ACCP10” (10 days plus 10 days); digital microscopic analysis using false color imaging. The crack regions in the control images (**A**,**B**) are marked (> crack <); in the “hCEM•ACCP10” samples no pronounced crack regions could be identified by light microscopy. Representative images are shown. The regions are analyzed both in y-axis and z-axis directions. Measurements are given in µm.

The change of the surface morphology of the cracked specimen (“hCEM”) as well as of a polyP-doped cement “hCEM•ACCP10” sample was analyzed with a surface structure imaging surface (Figure 10); representative samples are shown. The specimens were analyzed after an aging/maturation time of 10 days, followed by a post-incubation period of 10 days. In the control specimens, “hCEM” (Figure 10A,B), the cracks are still visible as > 400 µm deep fissures which are marked. In contrast, if the “hCEM•ACCP10” specimens were kept in water for additional 10 days (post-incubation period) the cracks disappeared and <200 µm small slots (in maximum) remained (Figure 10 C,D).

### 2.10. Mechanical Properties within Resealed Microcracks

**Figure 11:**
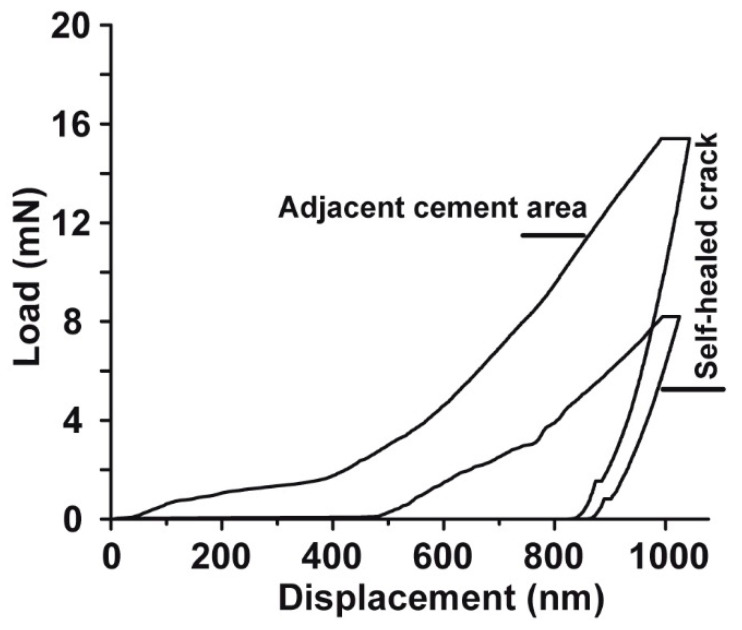
Hardness measurement within the self-healed crack area and the adjacent non-cracked area of “hCEM•ACCP10” cement samples. Samples were processed as follows; 10 days aging/maturation—introduction of cracks—10 days post-incubation. The nanoindentation measurements in the non-cracked cement area were performed at least 500 µm away from the cracked area. From the load/displacement curves the hardness and the elasticity (reduced Young’s modulus) were determined.

Nanoindentation measurements were performed at the surface plains of “ACCP10” containing cement samples, “hCEM•ACCP10”, both at repaired (self-healed) and at adjacent non-cracked sites. Representative load/displacement curves for both sites are shown in Figure 11. The surface plain of adjacent non-cracked “hCEM•ACCP10” is significantly harder, compared to the surface of self-healed cracks. This conclusion originates from the load/displacement curves. With respect to the values of the crack adjacent areas the load is about 100% higher and thus the material is harder if compared to the repaired concrete. Accordingly, the measured hardness value for the adjacent non-cracked surface area is distinctly higher with 0.79 ± 0.18 GPa (*n* = 48), compared with the self-healed crack area with 0.44 ± 0.12 GPa (*n* = 48). Interestingly, the self-healed crack surface area is slightly, but not significantly, more elastic, with a reduced Young’s modulus of 31.28 ± 7.97 GPa (*n* = 48) compared to the values measured for the adjacent non-cracked surface areas (32.97 ± 11.52 GPa; *n* = 48).

### 2.11. Analysis of Material Oozed into Cracks

**Figure 12:**
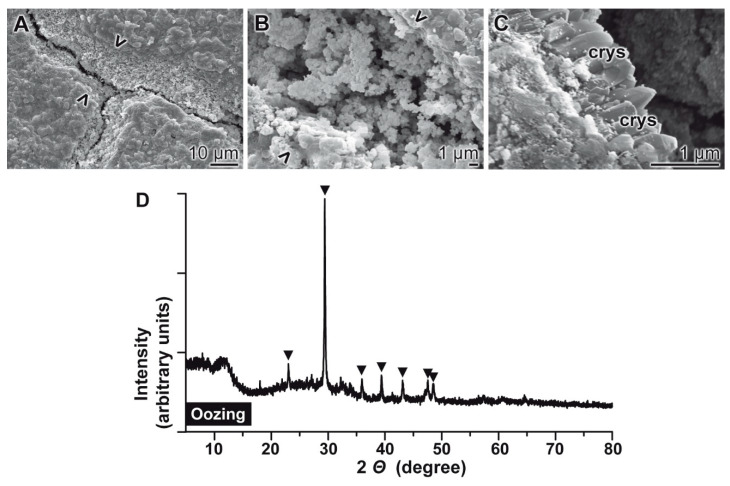
Microscopic analysis (ESEM) of the material oozed into cracks introduced into “hCEM•ACCP10” cement (**A–C**). Samples were treated as follows; 10-days aging/maturation—insertion of cracks—10 days post-incubation. The original borders of the cracks are marked (> <). (**D**) The grainy material formed within the cracks was scraped out of the healing site and analyzed by XRD. The signals characteristic for calcite are highlighted (▼). The crystals (crys) visible in ESEM can originate from calcite or from ettringite.

The material filled into cracks that had been introduced into “ACCP10” containing cement samples, “hCEM•ACCP10”, was analyzed both by ESEM and by XRD (Figure 12). The samples were aged for 10 days; then the cracks were introduced. Subsequently the samples were post-incubated in water for additional 10 days. During this period grainy particles dissolved and re-precipitated into the existing crack regions and sealed the defects (Figure 12A–C). At least some images taken at higher magnifications suggested that the grains in the cracks are decorated with calcite crystals (Figure 12C). The latter suggestion was confirmed by XRD analysis. The signals characteristic for calcite were detected (Figure 12D).

## 3. Discussion

As outlined in the introduction, the major rehydration product accumulated in smaller cracks in cement is calcium silicate hydrate (C–S–H) [7]. One of the major components contributing to the self-healing of these defects is CaCO_3_ [10]. In this study, we show that addition of ACC, amorphous CaCO_3_, added to the cement starting material, substantially induce the self-healing process in microcracks. As the major reaction deposit in the crack after a healing period for 10 days, while submersing in water, calcite crystals appeared. This material was absent in the cracks introduced into the controls, the polyP-free “hCEM”, material.

As an additive we selected ACC, stabilized with 10% (*w/w*) polyP. The soluble Na–polyP was used as the starting material for the fabrication of “ACCP10” together with CaCO_3_ [15]. A stoichiometric ratio between Ca^2+^ and CO_3_^2^^−^ of 1:1 was used, which results in a formation of crystalline calcite [15]. However, after addition of Na–polyP with a chain length of 40 P_i_ units to the Ca^2+^ and CO_3_^2^^−^–containing reaction mixture, amorphous “ACCP10” precipitated, as deduced from XRD studies. The spheric particles with a diameter of ≈20 nm formed had a low solubility in water with a solubility constant (*K_sp_*) of 3.3 × 10^−7^ (unpublished) at a pH of 7.5 to pH 10; transfer into dilute acid turned the material to a readily soluble salt. After addition of this material, “ACCP10”, to “CEM”, followed by transfer into an aqueous environment and leaving the specimens in a humid chamber for 10 days caused the formation of crystal structures, not seen in the “hCEM” controls. Based on published data it is safely to assume that those crystals are calcitic [19]. This result is also taken as an evidence that, in the otherwise alkaline “hCEM•ACCP10” cement, the ingredient “ACCP10” undergoes dissolution and subsequent transformation into calcite. This process is surely accelerated by abundantly present bacteria in the concrete and also in the air and the water. Bacteria exist in air with a density of ∼10^5^ m^–3^ [23]. They expose their cell wall on the enzyme alkaline phosphatase (ALP) that degrades/hydrolyzes polyP to inorganic phosphate [24] and by that removes this polymer, which stabilizes the amorphous state of Ca-carbonate. In the environment existing around the concrete, amorphous Ca-carbonate is readily transformed to vaterite/aragonite and finally to calcite [25]. This reaction is solely thermodynamically driven and does not need the co-assistance of an enzyme.

**Figure 13:**
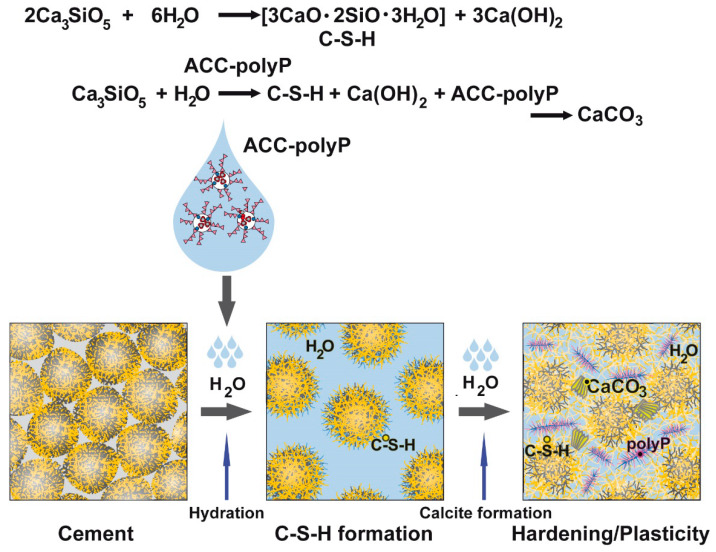
Schematic outline of the process of calcite formation in cement containing “ACCP10”. Both in the absence of “ACCP10”, in “hCEM” and in its presence, in “hCEM•ACCP10”, Ca_3_SiO_5_ undergoes a transformation into C–S–H in water under the release of calcium hydroxide turning the material into an alkaline paste. In the presence of “ACCP10” [ACC-polyP], in “hCEM•ACCP10”, calcite is additionally formed, turning the cement into a hard and a likewise more flexible material.

During this process, the amount of Ca(OH)_2_ decreases, but the percentage of C–S–H did not change, as checked by FTIR and also by XRD. The carbonation process of the existing Ca(OH)_2_ is driven by CO_2_ from the air [26]. Following published data, it can be assumed that during hydration of “hCEM•ACCP10” the tricalcium silicate (C_3_S) particles undergo transformation into C–S–H and release Ca(OH)_2_ [27]. In this reaction mixture, the conversion of “ACCP10” into calcite is facilitated (see below). Based on these reaction steps the formation of additional calcite from “ACCP10” under alkaline conditions can be sketched (Figure 13). As shown in this study, the additional calcite formed from “ACCP10” substantially contributes to the self-healing of cracks introduced into concrete, in addition to autonomous self-healing by Ca(OH)_2_ and CO_2_ (air).

In comparison to the control cement, “hCEM”, the ACC-containing material, “hCEM•ACCP10”, becomes somewhat less hard, but gains more flexibility/elasticity. These properties already suggests that the ACC-supplemented cement binder acquires hybrid/two-phase-like characteristics combining high tensile strength (strength at break) with a high modulus of elasticity (resistance to stretch and deformation prior to break) [28]. In the central part of the study data are presented that highlight the remarkable self-healing property of the “hCEM•ACCP10”. While in the controls, lacking ACC, no change in the morphology of the cracks can be microscopically identified, an almost complete sealing/healing of the cracks in the “hCEM•ACCP10” samples can be observed. The hardness of the crack-adjacent areas remained higher than that of the material migrated into the crack. However, the closing of the cracks with grain-like material originating from the adjacent cement area is exceptional, even if compared with bacteria-induced healing studies [29]. It is unlikely that addition of “ACCP10” to the cement can cause the generation of new cracks into the concrete because of two reasons. The “ACCP10” particles contain within their Ca-carbonate deposits also polyP, which most likely prevents a fast transformation of the amorphous phase of Ca-carbonate into a crystalline phase. During the aging process of the cement, the bacteria that co-exist with the “ACCP10” particles in the cement, with their alkaline phosphatase on their cell surfaces, dissolve the polymer polyP and allow a crystallization of Ca-carbonate to calcite [12]. The bacteria will initiate first, the enzymatic dissolution of the polyP in the particles and second, simultaneously, the formation of a polyP coacervate around the amorphous Ca-carbonate, as disclosed recently [13]. As a result, a core-shell arrangement is created that forms a hybrid entity between a very flexible coacervate and the more rigid Ca-carbonate (calcite) crystals.

## 4. Materials and Methods

### 4.1. Materials

Na–polyPhosphate (Na–polyP) with an average chain length of 40 phosphate (P_i_) units was purchased from Chemische Fabrik Budenheim (Budenheim; Germany). The Portland cement, CEM I 42.5 R, was a gift of the HeidelbergCement AG (Mainz; Germany-Dr. Ulrich Schneider). Analysis of the batch used (LN 43,308/001) revealed the following composition: 63.6% Ca_3_SiO_5_ (weight%), 1.6% α-Ca_2_SiO_4_, 4.3% β-Ca_2_SiO_4_, 6.4% aluminate [Ca_3_Al_2_O_6_] (cubic and orthorhombic), 12.6% ferrit [Ca_2_(Al,Fe)_2_O_5_], 1.9% K_2_SO_4_, 0.5% MgO, 0.3% CaO, 1.5% Ca(OH)_2_, 2.6% CaSO_4_, 2.7% CaSO_4_•0.5 H_2_O, 0.8% CaCO_3_, 0.4% SiO_2_, 0.6% CaMg(CO_3_)_2_.

### 4.2. Preparation of Amorphous Ca-Carbonate Microparticles

Amorphous calcium carbonate (ACC) nanoparticles (ACC-NP) were prepared as previously described [15,30] by direct precipitation from aqueous solution (room temperature), through rapid mixing of a CaCl_2_•2H_2_O solution (#223506; Sigma-Aldrich, Taufkirchen, Germany) and a Na_2_CO_3_ solution (#85,195; Fluka-Sigma) at an equimolar concentration ratio between Ca^2+^ and CO_3_^2−^. In brief, 20 mL of 0.1 M NaOH was added to 1.05 g Na_2_CO_3_ and then diluted with 30 mL of deionized water. This solution was added to 50 mL of water containing 1.47 g CaCl_2_•2H_2_O. The CaCO_3_ precipitate was rinsed with acetone to dry the solid material. In one series of experiments a sample of the precipitate was taken after a short, 30 min precipitation process and inspected by electron microscopy.

To stabilize the amorphous phase of the CaCO_3_, Na–polyP was added during the precipitation process. In turn, 10% (*w/w*) of the polymer (0.1 g Na–polyP) was immediately added to the CaCl_2_•2H_2_O/Na_2_CO_3_ solution, prior to the CaCO_3_ precipitation. The suspension obtained was filtrated, washed with acetone and dried at room temperature. The samples were termed “ACCP10”.

### 4.3. Preparation of Cement Samples

The unhydrated cement was termed “CEM”. The CEM I 42.5 R cement, 100 g, was mixed with 38 g of distilled water. This sample of hydrated cement was termed “hCEM“. In order to ensure a homogenous distribution of the solid particles in the cement, a longer blending time than usually used [31] with 30 min was chosen. During this period, the temperature increased slightly to ≈28 °C. The ratio cement:water was kept constant during the procedures described here.

In separated series of experiments, the cement was supplemented with “ACCP10” as follows: CEM I 42.5 R (100 g) was supplemented with 1 g “ACCP10” and subsequently reacted with 38 g of water; and termed “hCEM•ACCP10”.

The pH in the cement slurry, after addition of water, remained at ≈12.5.

The cement samples were prepared in 5 cm × 2.5 cm × 2.5 cm plastic molds and allowed, if not mentioned otherwise, to harden for 10 days prior to analysis; during this period the specimens remained in a humidity chamber (humidity: ~20 g/m^3^) at room temperature (20 °C). Then the samples were transferred to room humidity (at 20 °C) for 1 day and used for the experiments.

### 4.4. Microscopic Analyses

Electron microscopy was performed either with a scanning electron microscope (SEM), a HITACHI SU8000 electron microscope (Hitachi, Krefeld, Germany) or with an environmental scanning electron microscope (ESEM), using an ESEM XL-30 apparatus (Philips, Eindhoven; Netherlands), as described [32]. Light microscopical images were taken with a VHX-600 Digital Microscope from Keyence (Neu-Isenburg, Germany). The corresponding program to analyze the surface morphology was likewise obtained from Keyence.

### 4.5. Fourier Transformed Infrared Spectroscopy

Fourier transformed infrared spectroscopy (FTIR) was performed with ground cement material using a micro-mill and applying an ATR (attenuated total reflectance)-FTIR spectroscope/Varian 660-IR spectrometer (Agilent, Santa Clara, CA, USA), fitted with a Golden Gate ATR unit (Specac, Orpington, UK), as described [33].

### 4.6. X-Ray Diffraction

X-ray powder diffraction (XRD) of dried powder samples were analyzed with a D8 Advance A25 diffractometer (Bruker, Billerica, MA, USA) connected with a monochromatic Cu-Kα radiation [34].

### 4.7. Compression Testing

The hardening of the cement material with and without the addition of 1% (*w/w*) polyP-stabilized ACC (“ACCP10”) was assessed by means of compression testing at the following time-points after mixing: 5 h, 1 day, 2 days and 4 days (curing in a humidity chamber at 20 °C as mentioned above). The tests were performed using a “MultiTest 2.5-xt Force Testing System” equipped with a 2500 N Load Cell unit (Mecmesin, Ltd., Slinfold, UK). The cement samples were loaded in longitudinal direction with a loading speed of 5 mm/min using a 14 mm load-button. For this, loads of 2.5 N (5 h), 25 N (1 day) or 250 N (2 and 4 days) were applied to the samples and kept stable for 60 s. Subsequently, an unloading period (0 N) followed for 300 s. The force–displacement-time data were continuously recorded at a frequency of 50 Hz using the Emperor XT Force software (Mecmesin, Ltd.).

### 4.8. Mechanical Toughness: 3-Point Bend Testing

The breaking loads of the cement samples were determined with a 3-point bending system (5940 Series system; Instron, Norwood, MA), following a previously described procedure [12]. The standardized spherical loading head (diameter of 18 mm) and defined cement samples (83 mm in length × 30 mm width × 10 mm height) were used. The samples were centrally placed on top of two prismatic bars which were placed in parallel with a 50 mm distance to each other. The load was applied with an acceleration of 5 mm/min to the center of the sample body until failure. For all measurements the parameters force, compressive deformation and process time were continuously recorded.

### 4.9. Microcrack Formation and Self-healing Analysis

Following a previously described protocol [12], the sample “hCEM•ACCP10” was selected, which were aged for 10 days. Then microcracks were placed into the cement blocks by five shock freeze–warming cycles with liquid nitrogen (−196 °C)–heating at 80 °C. The resulting diameters of the cracks were ≈15 to ≈66 µm (48 ± 14 µm; 25 determinations). Subsequently, the samples remained submersed in water whose level reached 3 mm below the top edge of the blocks. The samples remained like this for 10 days prior to microscopic inspection and further analyses.

### 4.10. Determination of Mechanical Properties within Self-healed Microcracks

For these studies, the nanoindentation approach was applied, since this technique is useful for the determination of local mechanical properties, e.g., surface hardness and the local Young’s modulus. The instrument NanoTest Vantage System (Micro Materials, Ltd., Wrexham, UK) was applied, equipped with a Berkovich diamond indenter [35,36]. For each sample at least 20 independent measurements were performed at different sites with the following setting; maximum depth limit 1 µm, loading/unloading speed 0.15 mN/s and hold at maximum load 30 s. The spacing between two adjacent indents was set to a minimum of 3 mm. The Martens hardness as well as the Young’s modulus was calculated as described [35]. The following parameters for the indenter diamond were set [37]; Poisson ratio ν = 0.07 and elastic modulus *E* = 1143 GPa. All calculations were performed with the Nano-Test Platform Four V.40.08 software package (Micro Materials).

### 4.11. Statistical Analysis

The values reported are the average ± standard deviations. Statistical analyses were performed with the one-way ANOVA test, by using SigmaStat 3.5 software (Dundas Software, Ltd., Toronto, ON, Canada). Values of *p* < 0.05 were considered statistically significant.

## 5. Conclusions

**Figure 14:**
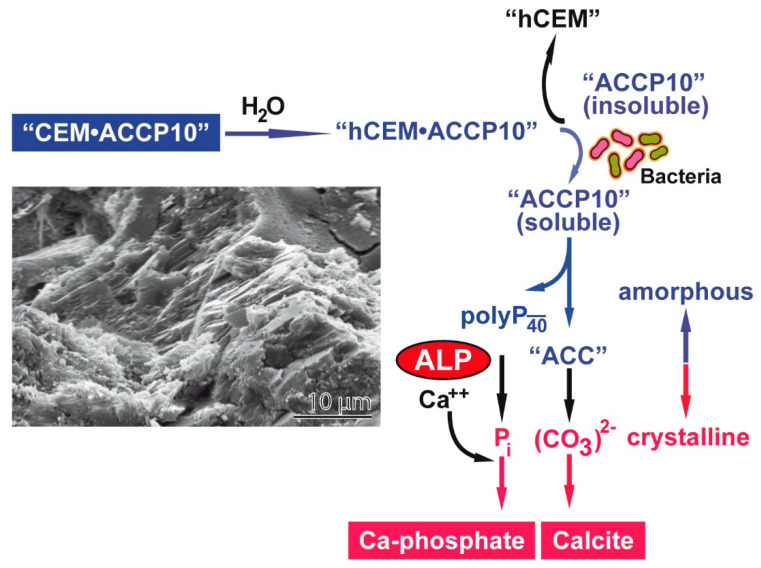
Schematic illustration of the reaction proceeding in the “ACCP10”-supplemented cement, “hCEM•ACCP10”, via formation of amorphous calcium carbonate and amorphous polyP to crystalline Ca-phosphate and crystalline Ca-carbonate (SEM image as insertion). The transition of the amorphous “ACCP10” in “hCEM•ACCP10” occurs after rendering “ACCP10” to a soluble phase. Subsequently, the bacterial alkaline phosphatase (ALP) enzymatically disintegrates the “ACCP10” particles to Ca^2+^ and P_i_ and initiates the process of crystal formation of Ca-phosphate and calcite.

The present study shows that addition of the bioinspired amorphous calcium carbonate, ACC, stabilized with Na–polyP of a chain length of 40 P_i_ units (“ACCP10”), substantially increases the self-healing property of Portland cement (“hCEM”). The reaction mechanism of this additive in the cement is quite unique (Figure 14). Prior to addition to the cement, the amorphous “ACCP10” particles are close to be insoluble in water at neutral or alkaline conditions. After addition to the cement (“hCEM•ACCP10”) the particles must be presumably dissolved since the subsequent reactions proceed under aqueous conditions. The conversion from the insoluble to the soluble phase—as described before [13]—is facilitated at the interphase between an inorganic and an organic phase like with bacteria residing in/on the cement. Also associated with bacteria is the ALP, an enzyme that degrades polyP to P_i_ [33,38]. Ca^2+^ is known to accumulate in cement and to leach out from this material [39]. In turn, P_i_ generated by the ALP, will readily precipitate with Ca^2+^ to Ca-phosphate at alkaline conditions [40] and form crystals [41]. The second component released in parallel from “ACCP10” is CO_3_^2^^−^, which will also immediately turn to a crystalline phase in the presence of Ca^2+^ [42]. This property of “ACCP10”, built from amorphous ACC and polyP, to form on the cement-bacteria interphase the crystalline materials required for self-healing in the cement, the calcium carbonate crystals, is remarkable and unique. At present, the commercial price for medium-sized polyP, as used in the studied cement formation, is comparatively high, in contrast to short-chain polyP, which is widely used in agriculture as a fertilizer, but could become affordable after upscaling for use as a cement additive.
